# The Arg1038Gly missense variant in the *NF1* gene causes a mild phenotype without neurofibromas

**DOI:** 10.1002/mgg3.616

**Published:** 2019-03-06

**Authors:** Eva Trevisson, Valeria Morbidoni, Monica Forzan, Cecilia Daolio, Valentina Fumini, Raffaele Parrozzani, Matteo Cassina, Edoardo Midena, Leonardo Salviati, Maurizio Clementi

**Affiliations:** ^1^ Department of Women’s and Children’s Health, Clinical Genetics Unit University of Padova Padova Italy; ^2^ Laboratorio di Genetica Clinica ed Epidemiologica Istituto di Ricerca Pediatrica, IRP Padova Italy; ^3^ Pediatric Unit, Carlo Poma Hospital Mantova Italy; ^4^ Department of Neurosciences University of Padova Padova Italy; ^5^ IRCCS–Fondazione Bietti Rome Italy

**Keywords:** Arg1038Gly, Genotype–phenotype correlation, missense mutation, neurofibromatosis type 1, NF1

## Abstract

**Background:**

Neurofibromatosis type 1 (NF1) is an autosomal dominant condition caused by inactivating mutations of the *NF1* gene. The wide allelic heterogeneity of this condition, with more than 3,000 pathogenic variants reported so far, is paralleled by its high clinical variability, which is observed even within the same family. The definition of genotype–phenotype correlations has been hampered by the complexity of the *NF1* gene and, although a few exceptions have been recognized, the clinical course remains unpredictable in most patients.

**Methods:**

Sequencing of NF1 in patients with cafè‐au‐lait spots identified the c.3112A>G variant. RNA analysis and a minigene assay were employed to investigate splicing.

**Results:**

Here we report a novel genotype–phenotype correlation in NF1: the identification of the missense variant NM_000267.3:c.3112A>G p.(Arg1038Gly) in seven individuals from two unrelated families with a mild phenotype. All the patients manifest cafè‐au‐lait spots without neurofibromas or other NF1–associated complications, and Noonan syndrome features in most cases. The missense variant was not previously reported in available databases, segregates with the phenotype and involves a highly conserved residue. Both a minigene assay and patient's RNA analysis excluded an effect on splicing.

**Conclusion:**

Our data support the correlation of the p.Arg1038Gly missense substitution with the cutaneous phenotype without neurofibromas or other complications. This finding may have relevant implications for patients and genetic counseling, but also to get insights into the function of neurofibromin.

## 1 INTRODUCTION

Neurofibromatosis type 1 (NF1) (MIM#162200) is an autosomal dominant disease caused by haploinsufficiency of the *NF1* gene (MIM #613113) (Gutmann et al., 2017). The prominent feature of this condition is its extremely variable phenotype, even within the same family (Ferner & Gutmann, 2013).

The *NF1* gene encodes for neurofibromin, a 2,838 amino acid long protein, which is involved in multiple cellular processes. The only functionally well–characterized domain is the GAP–related‐domain (GRD), which negatively regulates the RAS pathway. Other regions of the proteins are nevertheless relevant since they harbor pathogenic mutations (Gutmann, Parada, Silva, & Ratner, 2012).

The *NF1* gene is characterized by a wide mutational spectrum, with more than 3,000 genomic variants reported so far (Wallis et al., 2018). Approximately 10%–15% of patients harbor missense or inframe mutations: in this case validation may be problematic since less functional data are available for this protein. Importantly, a relevant fraction of exonic point mutations affects splicing, behaving like classical inactivating alleles (Pros et al., 2008).

The loss of heterozygosity of *NF1*, which was demonstrated in different affected tissues (Garcia‐Linares et al., 2011; De Schepper et al., 2008), is considered the driving pathogenetic mechanism in this condition, making impossible to predict the timing and nature of the somatic *NF1* mutations. However, there are some examples of relatively recurrent mutations associated with a partially concordant phenotype: (i) a more severe phenotype with a higher number of neurofibromas and a doubled relative risk to develop MPSNT in patients with a large deletion including *NF1* (Kehrer‐Sawatzki, Mautner, & Cooper, 2017); (ii) a milder phenotype without neurofibromas or other complications in patients with the inframe deletion delMet991 (Upadhyaya et al., 2007) a missense substitution involving the codon 1809 associated with cafe‐au‐lait spots (CALs) and Noonan–like features (Pinna et al., 2015; Rojnueangnit et al., 2015); (iii) a more severe phenotype with a higher risk of deep neurofibromas in patients with a missense mutation in one of the codons 844‐848 (Koczkowska et al., 2018).

**Figure 1:**
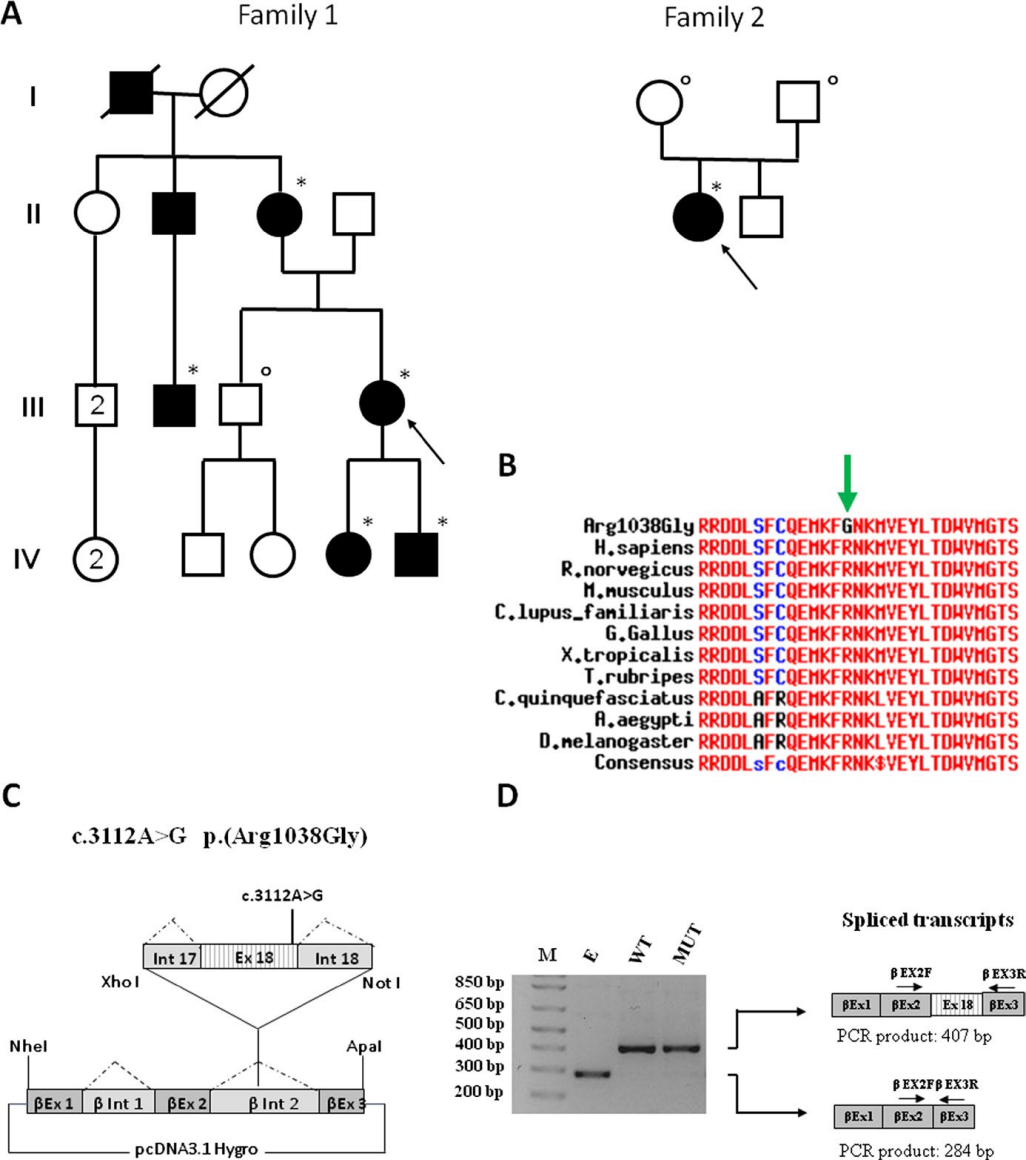
(a) Pedigree of the two families. Black symbols indicate individuals with cafè‐au‐lait spots. Subjects with * harbor the NM_000267.3:c.3112A>G variant of *NF1*, whereas individuals who were analyzed and do not show the mutation are marked with °. (b) The Arg1038Gly variant affects a highly conserved residue throughout evolution. (c) Schematic representation of the β‐globin minigene construct employed in this study. βEx1, βEx2, βEx3 refer to β‐globin exon 1, 2, and 3. βInt1, βInt2 refer to β‐globin intron 1 and 2. A fragment of 566 nucleotides including exon 18 with at least 100 bp of the flanking introns (Int 17 and Int 18) was amplified from patient III, 5 DNA with primers inserting the XhoI and NotI restrictions sites for the subsequent cloning into the pCDNA3.1‐Beta‐globin vector. One mutant and one wild‐type clone were retrieved for subsequent experiments. (d) Total RNA extracted from HeLa cells transfected with the wild‐type (WT), the mutant construct (MUT) and the empty vector (e) was retro‐transcribed and PCR‐amplified using primers specific for β‐globin exon 2 (β EX2F) and 3 (β EX3R) and separated by agarose gel electrophoresis. The mutant construct (MUT) showed the same splicing pattern as the control (WT).

In this work we report the missense mutation p.(Arg1038Gly) in the *NF1* gene segregating in seven patients from two unrelated families who manifest a mild NF1 and propose a novel genotype–phenotype correlation. Pedigrees of both families are displayed in Figure 1a, whereas the clinical features of the available family members are reported in Table 1.

**Table 1:** Clinical features of the seven patients from the two families harboring the p.Arg1038Gly variant

Clinical features	Family 1						Family 2
	IV, 5	IV,6	III,5	III,3	II,3	II,2	III,1
Sex	F	M	F	M	F	M	F
Age (years)	10	2	41	38	67	72	30
>5 CALs >1.5 cm^a^	+ (>0.5 cm)	+ (>0.5 cm)	+	+	+	+	+
Axillary/inguinal freckling	−	−	+ (groin only)	−	−	−	+
Lisch nodules	−	−	−	n.a.	n.a.	n.a.	−
Cutaneous neurofibromas	−	−	−	−	−	−	−
Symptomatic neurofibromas	−	−	−	−	−	−	−
Diffuse plexiform neurofibromas	−	−	−	−	−	−	−
Optic pathway glioma	−	−	−	−	−	−	− (UBOs at brain MRI)
Short stature	−	−	−	−	−	−	+
Macrocephaly	−	+	−	+	−	−	+
Pulmonary valve stenosis	−c	−c	−c	−	−	−	−c
Facial NS features^b^	+	+	+	−	+	−	+
Learning disabilities	−	n.a.	−	−	−	−	−

Note

For each specific sign: "–" means absent, "+" present, "n.a." = not available.

a In all patients CALs were diffuse.

b Facial Noonan Syndrome (NS) features include hypertelorism with down–slanting palpebral fissures.

c Normal cardiac ultrasound.

Ethical compliance: informed consent was obtained for all the patients.

Family 1 was referred to our outpatient clinic for multiple CALs. The proband (III,5) was a 41‐year old woman whose clinical history was unremarkable. Our physical examination revealed a normal head circumference (+0.4 *SD*) and showed the presence of more than 10 CALs with a diameter >1.5 cm, mostly at the trunk with rare freckling at the groins and some cherry spots. No cutaneous or subcutaneous neurofibromas could be detected and a detailed ophthalmological evaluation excluded the presence of NF1–related signs. Both her children, a 10‐year old girl (IV,5) and a 2‐year old boy (IV,6), who had normal growth and psychomotor development, showed more than 5 CALs with a diameter >0.5 cm without atypical freckling. Ophthalmological evaluations did not show any sign of NF1 and a cardiac US was normal. Their clinical evaluations revealed hypertelorism with mildly down–slanting palpebral fissures, and the boy also showed macrocephaly (+2.7 *SD*) with frontal bossing, posteriorly rotated ears and mild retrognathia.

CALs were present also in the proband's mother, her grandfather, the maternal uncle and the first–degree cousin (Figure 1a), neither of whom manifest neurofibromas or other signs of NF1 except for CALs.

Patient II,1 from family 2 is a 30–year old woman who was referred to our center at age 2 for the presence of multiple CALs. The clinical examination at that time revealed normal auxological parameters, hypertelorism, multiple CALs and rare axillary and inguinal freckling, without congenital plexiform neurofibromas, or skeletal anomalies. The subsequent follow‐up revealed a normal psychomotor development, failure to thrive (weight and height at the lower centiles) and a pubertal delay, but no other complications related to NF1 and normal ophthalmological evaluations. An asymptomatic 5‐cm subcutaneous nodule in the back was removed and resulted a lipoma. A brain MRI performed at 19 years was normal, except for the presence of UBOs at the level of left lenticular nucleus and the periventricular white matter. Her family history was unremarkable and her parents did not show cutaneous or ocular signs of NF1.

*NF1* and the other genes of the RAS pathway (*BRAF*, *CBL*, *CDC42*, *HRAS*, *KRAS*, *LZTR1*, *MAP2K1*, *MAP2K2*, *MAP3K8*, *MYST4*, *NRAS*, *PTPN11*, *RAF1*, *RASA2*, *RIT1*, *RRAS*, *SHOC2*, *SOS1*, *SOS2*, *SPRED1*, *SPRY1*) were sequenced on an Illumina MiSeq platform with an Agilent Haloplex kit (Santa Clara CA) with coverage >20× in >99% of the target regions.

Molecular analysis identified the heterozygous missense variant NM_000267.3:c.3112A>G p.(Arg1038Gly) in the *NF1* gene in patient (III,5) from Family 1, which segregated in all the affected members (Figure 1a). Sequencing of *SPRED1* and of the other genes mutated in RASopathies did not detect any pathogenetic variant and MLPA for *NF1*, which was performed with a commercial kit (MRC Holland, The Netherlands), excluded deletions or duplications. Phenotype was concordant in all family members and characterized by CALs in all patients with Noonan–like features in most cases.

The NM_000267.3:c.3112A>G allele was absent in the HGMD, LOVD, ExAC, GnomAD, or dbSNP databases and has been previously reported in a single unrelated patient fulfilling the diagnostic criteria for NF1 (Corsello et al., 2018) (unfortunately no clinical data are available for this patient). We therefore checked our in–house database of 732 NF1 patients and found the same variant in an unrelated individual (patient II,1 from family 2), who also displays a CALs–only phenotype. Her parents do not show clinical signs of NF1 and, accordingly, do not harbor the missense allele; parental identity was confirmed.

Since the c.3112A>G variant involves the penultimate nucleotide of exon 23 (exon 18 according to the NF1 Consortium Nomenclature) and in silico analyses with different software (Human Splicing Finder, Mutation Taster) predict a possible effect on splicing with the creation of an exonic ESS site, we analyzed cDNA from fresh blood in patient III, 5 as detailed elsewhere (Heim et al., 1995). Direct sequencing of the RT‐PCR amplicon containing exon 13‐27 did not detect any aberrant splicing. Since mutant mRNA may undergo NMRD, we also employed a minigene assay (Figure 1c) to exclude an effect on transcript maturation using a β‐globin hybrid construct as previously reported (Cassina et al., 2017). As showed in Figure 1d, cells transfected with the mutant minigene construct show the same splicing pattern as those expressing the WT plasmid, suggesting that the nucleotide change does not affect splice site recognition. Direct sequencing of the PCR fragment confirmed this finding. The novel *NF1* variant has been submitted to the LOVD database [www.lovd.nl/NF1 (DB‐ID NF1_002517)].

There are different pieces of evidence supporting the pathogenicity of this variant: it is extremely rare and it is predicted to cause a nonconservative amino acid change, resulting in the replacement of a positively charged arginine with a neutral glycine. This residue lays within a highly conserved region: arginine 1038 is conserved up to arthropods (Figure 1b) and a different missense substitution affecting the same codon (p.Arg1038Ser) was previously reported in a NF1 patient (Lee et al., 2006), underlying its relevant role for protein structure and/or function. Most of the prediction software tools (PolyPhen2, SIFT, Mutation Taster) we examined, including those integrating multiple tools such as CADD and REVEL (with score of 30 and 0.661, respectively) are in favor of a pathogenetic role of this substitution. Based on our studies on the transcript, the functional consequences of this mutation rely on the presence of the amino acid substitution on a normally spliced protein product. Population, in silico and clinical data (segregation with the cutaneous phenotype in multiple family members with cosegregation analysis of 1/32 according to Jarvik & Browning, 2016 and de novo appearance in a sporadic case) allow to classify this variant as pathogenic (class 5) according to the American College of Medical Genetics and Genomics Standards and Guidelines (Richards et al., 2015).

The effects on protein function are difficult to establish considering the lack of information about this domain of neurofibromin. It is possible that it exhibits a hypomorphic activity, similarly to other alleles that have been associated with a mild phenotype (Pinna et al., 2015; Upadhyaya et al., 2007). Arginine 1038 is located 160 amino acids upstream to the GRD domain of neurofibromin. Nevertheless, it may have functional consequences on the RAS pathway, since it might affect the interaction with downstream Ras‐effectors, as in the case of the R1809C mutation (Wallis et al., 2018). This hypothesis is intriguing since some of our patients display Noonan–like features, but needs functional confirmation.

Unlike the striking clinical variability usually observed within NF1 individuals, the phenotype of the patients harboring the c.3112A>G mutations is quite homogeneous (Table 1): all individuals display CALs, with freckling being present in 28%; none of them shows cutaneous neurofibromas (that are present in most NF1 patients >20 years) (Friedman & Birch, 1997) (Upadhyaya et al., 2007), symptomatic spinal or plexiform neurofibromas, ocular hamartomas or symptomatic OPG (brain MRI was performed only in one patient and showed UBOs). Similarly, learning disabilities were not reported in these patients. It should be noted that some clinical signs, such as deep asymptomatic neurofibromas, may be missed during clinical evaluations. Moreover, the absence of NF1–associated complications, particularly those with lower prevalence, must be confirmed in larger cohorts to draw definitive conclusions.

An increased prevalence of nontruncating mutations in NF1 patients with Noonan–like features was previously observed (De Luca et al., 2005), and another study hypothesized the role of nontruncating mutations on the NF1 cardiac phenotype (Ben‐Shachar et al., 2013). None of the patients harboring the p.Arg1038Gly mutations displays pulmonary stenosis (or other cardiac manifestations), but at least five individuals show facial features resembling the Noonan phenotype. This clinical association may be underestimated in our series, since most patients have been examined in adulthood, where Noonan–associated facial features are harder to detect.

Interestingly, some of the mutations reported in exon 17 and exon 18 have been associated with a phenotype overlapping with other Ras–MAPK disorders: the inframe deletion in exon 17 (Upadhyaya et al., 2007) was related to a higher incidence of pulmonary stenosis without neurofibromas. The p.(Met1035Arg) allele was described in a patient with a Leopard–like phenotype (Wu et al., 1996). LOVD and HGMD report thirteen missense substitutions in exon 18 (including one benign variant): except for four cases (Fauth et al., 2009) (Kluwe, Friedrich, Peiper, Friedman, & Mautner, 2003; Xu, Yang, Hu, & Li, 2014), most of them are classified as variants of unknown significance and unfortunately there are no clinical data, nor it is clear whether RNA maturation is affected, as for the c.3114G>T p.(Arg1038Ser) (Lee et al., 2006) allele. Two missense variants in the initial part of exon 18, p.(Leu1015Pro) (Kluwe et al., 2003) and p.(Cys1016Arg) (Fauth et al., 2009), have been associated with a severe phenotype with the development of MPSNT.

Our findings demonstrated the pathogenicity of the c.3112A>G p.Arg1038Gly variant and support its association with a cutaneous phenotype with lower frequency of NF1–associated complications and Noonan–like features in some individuals. Further studies on larger NF1 cohorts for which clinical data are available are required to corroborate this novel genotype–phenotype correlation. The confirmation of this hypothesis will not only facilitate clinicians in genetic counseling and surveillance programs, but will also help in defining the role of neurofibromin within cells.

## CONFLICT OF INTEREST

The authors declare no conflict of interest.
